# 3-{[(4*Z*)-1,2-Dimethyl-5-oxoimidazol-4-yl­idene]meth­yl}-4-hy­droxy­benzonitrile

**DOI:** 10.1107/S1600536812007532

**Published:** 2012-02-29

**Authors:** Hsing-Yang Tsai, Ming-Jen Chang, Tzu-Chien Fang, Ming-Hui Luo, Kew-Yu Chen

**Affiliations:** a Department of Chemical Engineering, Feng Chia University, 40724 Taichung, Taiwan

## Abstract

In the title compound, C_13_H_11_N_3_O_2_, an intra­molecular O—H⋯N hydrogen bond generates an *S*(7) ring. The dihedral angle between the mean plane of the benzene ring and the imidazolidinone ring is 3.05 (2)°. In the crystal, inversion-related mol­ecules are linked by dual C—H⋯O_carbon­yl_ hydrogen bonds to form a dimer with an *R*_2_^2^(14) graph-set motif. A C—H⋯O_hy­droxy_ inter­action links pairs of mol­ecules into another type of cyclic dimer with an *R*_2_^2^(18) motif. The mol­ecules are further linked by C—H⋯N inter­actions to form layers parallel to (001). Offset π–π stacking [3.3877 (8) Å] is observed in the crystal structure, with an inter­planar spacing between the planes of neighboring benzene rings of 3.444 (1) Å.

## Related literature

For the spectroscopy and preparation of the title compound, see: Chuang *et al.* (2011 ▶). For the applications of proton-transfer dyes, see: Chen & Pang (2010 ▶); Gryko *et al.* (2010 ▶); Han *et al.* (2010 ▶); Helal *et al.* (2010 ▶); Ikeda *et al.* (2010 ▶); Ito *et al.* (2011 ▶); Lim *et al.* (2011 ▶); Lins *et al.* (2010 ▶); Maupin *et al.* (2011 ▶); Santos *et al.* (2011 ▶); Tang *et al.* (2011 ▶). For a related structure, see: Chen *et al.* (2007 ▶). For graph-set notation of hydrogen bonds, see: Bernstein *et al.* (1995 ▶).
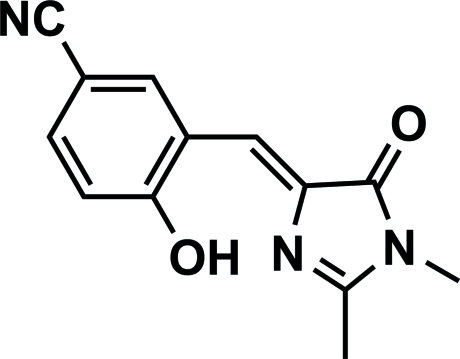


## Experimental

### 

#### Crystal data

C_13_H_11_N_3_O_2_
*M* *_r_* = 241.25
Monoclinic, 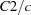

*a* = 24.9655 (10) Å
*b* = 3.8349 (1) Å
*c* = 26.7584 (10) Å
β = 115.488 (5)°
*V* = 2312.52 (17) Å^3^
*Z* = 8
Mo *K*α radiation
μ = 0.10 mm^−1^
*T* = 150 K
0.24 × 0.2 × 0.15 mm

#### Data collection

Bruker SMART CCD diffractometer
Absorption correction: multi-scan (*SADABS*; Bruker, 2001 ▶) *T*_min_ = 0.811, *T*_max_ = 0.999
15085 measured reflections
2048 independent reflections
1364 reflections with *I* > 2σ(*I*)
*R*_int_ = 0.067

#### Refinement

*R*[*F* ^2^ > 2σ(*F* ^2^)] = 0.042
*wR*(*F* ^2^) = 0.112
*S* = 0.94
2048 reflections
166 parameters
H-atom parameters constrained
Δρ_max_ = 0.25 e Å^−3^
Δρ_min_ = −0.29 e Å^−3^

### 

Data collection: *SMART* (Bruker, 2001 ▶); cell refinement: *SAINT* (Bruker, 2001 ▶); data reduction: *SAINT*; program(s) used to solve structure: *SHELXS97* (Sheldrick, 2008 ▶); program(s) used to refine structure: *SHELXL97* (Sheldrick, 2008 ▶); molecular graphics: *ORTEP-3 for Windows* (Farrugia, 1997 ▶); software used to prepare material for publication: *WinGX* (Farrugia, 1999 ▶).

## Supplementary Material

Crystal structure: contains datablock(s) I, global. DOI: 10.1107/S1600536812007532/zl2452sup1.cif

Structure factors: contains datablock(s) I. DOI: 10.1107/S1600536812007532/zl2452Isup2.hkl

Supplementary material file. DOI: 10.1107/S1600536812007532/zl2452Isup3.cml

Additional supplementary materials:  crystallographic information; 3D view; checkCIF report

## Figures and Tables

**Table 1:** Hydrogen-bond geometry (Å, °)

*D*—H⋯*A*	*D*—H	H⋯*A*	*D*⋯*A*	*D*—H⋯*A*
O2—H2⋯N2	0.82	1.85	2.591 (2)	150
C12—H12⋯O1^i^	0.93	2.47	3.262 (2)	143
C5—H5*C*⋯O2^ii^	0.96	2.67	3.566 (3)	156
C4—H4*A*⋯N3^iii^	0.96	2.63	3.436 (3)	142
C5—H5*A*⋯N3^iv^	0.96	2.64	3.586 (3)	169

Symmetry codes: (i) 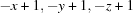; (ii) 
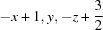
; (iii) 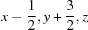; (iv) 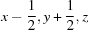.

**Fig. 1.**
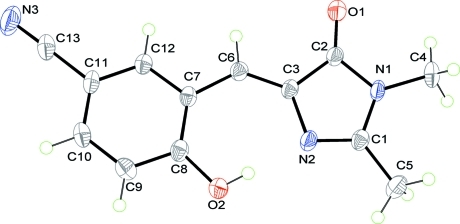
The molecular structure of the title compound, showing 50% probability displacement ellipsoids.

**Fig. 2.**
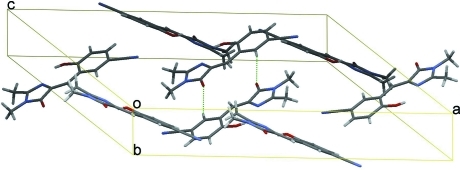
A section of the crystal packing of the title compound, viewed down the c axis. Green dashed lines denote the intermolecular C12—H12···O1 hydrogen bonds [symmetry code: -x + 1, -y + 1, -z + 1].

## supplementary crystallographic information

### Comment

The excited-state intramolecular proton transfer (ESIPT) reaction of the title compound has been investigated recently (Chuang *et al.*, 2011), which incorporates transfer of a hydroxy proton to the imine nitrogen through an intramolecular seven-membered-ring hydrogen-bonding system. The proton transfer dyes have found many important applications. Prototypical examples are probes for solvation dynamics (Chen & Pang, 2010; Lins *et al.*, 2010) and biological environments (Lim *et al.*, 2011; Maupin *et al.*, 2011), fluorescence microscopy imaging (Santos *et al.*, 2011), near-infrared fluorescent dyes (Ikeda *et al.*, 2010), photochromic materials (Ito *et al.*, 2011), chemosensors (Han *et al.*, 2010; Helal *et al.*, 2010) and recent application in the field of organic light emitting devices (Gryko *et al.*, 2010; Tang *et al.*, 2011).

The molecular structure of the title compound is shown in Fig. 1. As expected, the molecule possesses an intramolecular O—H···N hydrogen bond, which generates an S(7) ring (Chen *et al.*, 2007). The dihedral angle between the mean plane of the benzene ring and the imidazolidinone ring is 3.05 (2)°. In the crystal (Fig. 2), inversion-related molecules are linked by pairs of C12—H12···O1 hydrogen bonds, forming a cyclic dimer with an *R*_2_^2^(14) graph-set motif, Fig. 2 (Bernstein *et al.*, 1995). In addition, the C5—H5C···O2 interaction links a pair of molecules into another type of cyclic dimer with an *R*_2_^2^(18) graph-set motif. Molecules are further stabilized by intermolecular C—H···N interactions involving the methyl groups of C4 and C5 to form layers parallel to (001). See Table 1 for numerical details of the hdrogen bonds and symmetry operators. Offset π–π stacking is observed in the crystal structure with an interplanar spacing between planes of neighboring benzene rings of 3.444 (1) Å. The closest centroid–centroid distance [symmetry code: *x*, -1 + *y*, *z*] is 4.8350 (12) Å (*Cg*1 and *Cg*2 are the centroids of the N1/N2/C1–C3 and C7–C12 rings, respectively).

### Experimental

The title compound was synthesized according to the literature (Chuang *et al.*, 2011). Yellow needle-shaped crystals suitable for the crystallographic studies reported here were isolated over a period of six weeks by slow evaporation from a chloroform solution.

### Refinement

H atoms bonded to O and C atoms were located in a difference electron density map. In the final model, H atoms were repositioned geometrically and refined using a riding model [C—H = 0.93 Å for C_sp2_ H atoms, 0.96 for C_sp3_ H atoms, 0.82 Å for hydroxy H atoms and *U*_iso_(H) = 1.2 (C_sp2_) or 1.5 (C_sp3_, O) *U*_eq_(C/O)]. The hydroxy H atoms and C_sp3_ H atoms were allowed to rotate but not to tip to best fit the experimental electron density.

### Crystal data

**Table:**

C_13_H_11_N_3_O_2_	*F*(000) = 1008
*M**_r_* = 241.25	*D*_x_ = 1.386 Mg m^−^^3^
Monoclinic, *C*2/*c*	Mo *K*α radiation, λ = 0.71073 Å
Hall symbol: -C 2yc	Cell parameters from 3648 reflections
*a* = 24.9655 (10) Å	θ = 3.1–26.9°
*b* = 3.8349 (1) Å	µ = 0.10 mm^−^^1^
*c* = 26.7584 (10) Å	*T* = 150 K
β = 115.488 (5)°	Prism, colourless
*V* = 2312.52 (17) Å^3^	0.24 × 0.2 × 0.15 mm
*Z* = 8	

### Data collection

**Table:**

Bruker SMART CCD diffractometer	2048 independent reflections
Radiation source: fine-focus sealed tube	1364 reflections with *I* > 2σ(*I*)
Graphite monochromator	*R*_int_ = 0.067
φ and ω scans	θ_max_ = 25.0°, θ_min_ = 1.7°
Absorption correction: multi-scan (*SADABS*; Bruker, 2001)	*h* = −28→28
*T*_min_ = 0.811, *T*_max_ = 0.999	*k* = −4→4
15085 measured reflections	*l* = −31→31

### Refinement

**Table:**

Refinement on *F*^2^	Primary atom site location: structure-invariant direct methods
Least-squares matrix: full	Secondary atom site location: difference Fourier map
*R*[*F*^2^ > 2σ(*F*^2^)] = 0.042	Hydrogen site location: inferred from neighbouring sites
*wR*(*F*^2^) = 0.112	H-atom parameters constrained
*S* = 0.94	*w* = 1/[σ^2^(*F*_o_^2^) + (0.0695*P*)^2^] where *P* = (*F*_o_^2^ + 2*F*_c_^2^)/3
2048 reflections	(Δ/σ)_max_ = 0.001
166 parameters	Δρ_max_ = 0.25 e Å^−^^3^
0 restraints	Δρ_min_ = −0.29 e Å^−^^3^

### Special details

**Table:**

Geometry. All e.s.d.'s (except the e.s.d. in the dihedral angle between two l.s. planes) are estimated using the full covariance matrix. The cell e.s.d.'s are taken into account individually in the estimation of e.s.d.'s in distances, angles and torsion angles; correlations between e.s.d.'s in cell parameters are only used when they are defined by crystal symmetry. An approximate (isotropic) treatment of cell e.s.d.'s is used for estimating e.s.d.'s involving l.s. planes.
Refinement. Refinement of *F*^2^ against ALL reflections. The weighted *R*-factor *wR* and goodness of fit *S* are based on *F*^2^, conventional *R*-factors *R* are based on *F*, with *F* set to zero for negative *F*^2^. The threshold expression of *F*^2^ > σ(*F*^2^) is used only for calculating *R*-factors(gt) *etc*. and is not relevant to the choice of reflections for refinement. *R*-factors based on *F*^2^ are statistically about twice as large as those based on *F*, and *R*-factors based on ALL data will be even larger.

### Fractional atomic coordinates and isotropic or equivalent isotropic displacement parameters (Å^2^)

**Table:**

	*x*	*y*	*z*	*U*_iso_*/*U*_eq_	
O1	0.41612 (5)	0.5235 (4)	0.49910 (5)	0.0339 (4)	
O2	0.56473 (6)	0.0638 (3)	0.71292 (5)	0.0339 (4)	
H2	0.5299	0.0855	0.6907	0.051*	
N1	0.38975 (6)	0.5881 (4)	0.57176 (6)	0.0246 (4)	
N2	0.46849 (6)	0.3562 (4)	0.64190 (6)	0.0251 (4)	
N3	0.74916 (8)	−0.3544 (5)	0.59904 (8)	0.0484 (6)	
C1	0.41673 (8)	0.5046 (5)	0.62728 (8)	0.0245 (5)	
C2	0.42684 (8)	0.4890 (5)	0.54770 (8)	0.0235 (5)	
C3	0.47832 (7)	0.3369 (5)	0.59435 (7)	0.0221 (4)	
C4	0.33058 (8)	0.7365 (5)	0.54035 (8)	0.0331 (5)	
H4A	0.3244	0.9241	0.5610	0.050*	
H4B	0.3012	0.5594	0.5339	0.050*	
H4C	0.3274	0.8233	0.5055	0.050*	
C5	0.38931 (9)	0.5833 (5)	0.66510 (8)	0.0347 (5)	
H5A	0.3499	0.4879	0.6503	0.052*	
H5B	0.3874	0.8314	0.6689	0.052*	
H5C	0.4127	0.4818	0.7007	0.052*	
C6	0.52658 (8)	0.2123 (5)	0.58951 (7)	0.0231 (5)	
H6	0.5242	0.2299	0.5539	0.028*	
C7	0.58150 (7)	0.0561 (4)	0.62927 (7)	0.0212 (5)	
C8	0.59742 (8)	−0.0138 (5)	0.68578 (8)	0.0247 (5)	
C9	0.65172 (8)	−0.1766 (5)	0.71763 (8)	0.0291 (5)	
H9	0.6619	−0.2264	0.7546	0.035*	
C10	0.69024 (8)	−0.2644 (5)	0.69540 (8)	0.0277 (5)	
H10	0.7262	−0.3715	0.7173	0.033*	
C11	0.67552 (8)	−0.1933 (5)	0.64034 (8)	0.0251 (5)	
C12	0.62173 (8)	−0.0365 (5)	0.60796 (8)	0.0241 (5)	
H12	0.6121	0.0087	0.5709	0.029*	
C13	0.71618 (8)	−0.2836 (5)	0.61677 (8)	0.0323 (5)	

### Atomic displacement parameters (Å^2^)

**Table:**

	*U*^11^	*U*^22^	*U*^33^	*U*^12^	*U*^13^	*U*^23^
O1	0.0263 (8)	0.0464 (9)	0.0299 (9)	0.0084 (6)	0.0128 (6)	0.0040 (7)
O2	0.0225 (7)	0.0537 (10)	0.0266 (8)	0.0042 (6)	0.0116 (6)	0.0030 (7)
N1	0.0157 (8)	0.0296 (10)	0.0305 (9)	0.0027 (7)	0.0119 (7)	−0.0007 (8)
N2	0.0192 (9)	0.0305 (10)	0.0287 (9)	−0.0006 (7)	0.0132 (7)	−0.0024 (8)
N3	0.0312 (11)	0.0575 (14)	0.0624 (14)	0.0070 (9)	0.0257 (10)	−0.0083 (11)
C1	0.0210 (11)	0.0246 (11)	0.0303 (11)	−0.0034 (9)	0.0133 (9)	−0.0035 (9)
C2	0.0198 (10)	0.0266 (11)	0.0261 (12)	−0.0005 (8)	0.0118 (9)	−0.0009 (9)
C3	0.0189 (10)	0.0239 (11)	0.0259 (10)	−0.0016 (8)	0.0118 (8)	−0.0012 (8)
C4	0.0192 (10)	0.0351 (12)	0.0439 (13)	0.0077 (9)	0.0124 (10)	0.0028 (10)
C5	0.0338 (12)	0.0395 (13)	0.0385 (13)	0.0029 (10)	0.0230 (11)	−0.0006 (10)
C6	0.0205 (10)	0.0249 (11)	0.0249 (10)	−0.0020 (8)	0.0108 (8)	−0.0015 (9)
C7	0.0172 (10)	0.0187 (10)	0.0279 (11)	−0.0028 (8)	0.0099 (9)	−0.0025 (8)
C8	0.0185 (10)	0.0256 (11)	0.0314 (12)	−0.0036 (8)	0.0119 (9)	−0.0027 (9)
C9	0.0248 (11)	0.0308 (12)	0.0273 (11)	−0.0016 (9)	0.0070 (9)	0.0026 (9)
C10	0.0181 (10)	0.0244 (11)	0.0363 (12)	0.0012 (8)	0.0075 (9)	0.0005 (9)
C11	0.0180 (10)	0.0211 (10)	0.0368 (12)	0.0005 (8)	0.0124 (9)	−0.0039 (9)
C12	0.0206 (10)	0.0245 (11)	0.0281 (11)	0.0003 (8)	0.0115 (9)	−0.0008 (9)
C13	0.0209 (11)	0.0307 (12)	0.0425 (13)	0.0032 (9)	0.0109 (10)	−0.0009 (10)

### Geometric parameters (Å, º)

**Table:**

O1—C2	1.217 (2)	C5—H5A	0.9600
O2—C8	1.339 (2)	C5—H5B	0.9600
O2—H2	0.8200	C5—H5C	0.9600
N1—C1	1.379 (2)	C6—C7	1.454 (2)
N1—C2	1.389 (2)	C6—H6	0.9300
N1—C4	1.464 (2)	C7—C12	1.397 (2)
N2—C1	1.308 (2)	C7—C8	1.414 (2)
N2—C3	1.398 (2)	C8—C9	1.400 (3)
N3—C13	1.146 (2)	C9—C10	1.373 (2)
C1—C5	1.476 (2)	C9—H9	0.9300
C2—C3	1.473 (2)	C10—C11	1.383 (3)
C3—C6	1.353 (2)	C10—H10	0.9300
C4—H4A	0.9600	C11—C12	1.384 (3)
C4—H4B	0.9600	C11—C13	1.449 (3)
C4—H4C	0.9600	C12—H12	0.9300
			
C8—O2—H2	109.5	H5A—C5—H5C	109.5
C1—N1—C2	108.77 (15)	H5B—C5—H5C	109.5
C1—N1—C4	127.81 (15)	C3—C6—C7	132.33 (17)
C2—N1—C4	123.33 (15)	C3—C6—H6	113.8
C1—N2—C3	106.78 (15)	C7—C6—H6	113.8
N2—C1—N1	112.63 (16)	C12—C7—C8	117.87 (17)
N2—C1—C5	125.00 (17)	C12—C7—C6	115.02 (16)
N1—C1—C5	122.36 (16)	C8—C7—C6	127.10 (16)
O1—C2—N1	125.56 (17)	O2—C8—C9	115.19 (17)
O1—C2—C3	131.25 (17)	O2—C8—C7	125.55 (17)
N1—C2—C3	103.19 (15)	C9—C8—C7	119.26 (17)
C6—C3—N2	128.19 (17)	C10—C9—C8	121.40 (18)
C6—C3—C2	123.15 (17)	C10—C9—H9	119.3
N2—C3—C2	108.63 (14)	C8—C9—H9	119.3
N1—C4—H4A	109.5	C9—C10—C11	119.90 (17)
N1—C4—H4B	109.5	C9—C10—H10	120.1
H4A—C4—H4B	109.5	C11—C10—H10	120.1
N1—C4—H4C	109.5	C10—C11—C12	119.61 (17)
H4A—C4—H4C	109.5	C10—C11—C13	120.11 (17)
H4B—C4—H4C	109.5	C12—C11—C13	120.28 (18)
C1—C5—H5A	109.5	C11—C12—C7	121.95 (18)
C1—C5—H5B	109.5	C11—C12—H12	119.0
H5A—C5—H5B	109.5	C7—C12—H12	119.0
C1—C5—H5C	109.5	N3—C13—C11	178.8 (2)

### Hydrogen-bond geometry (Å, º)

**Table:**

*D*—H···*A*	*D*—H	H···*A*	*D*···*A*	*D*—H···*A*
O2—H2···N2	0.82	1.85	2.591 (2)	150
C12—H12···O1^i^	0.93	2.47	3.262 (2)	143
C5—H5*C*···O2^ii^	0.96	2.67	3.566 (3)	156
C4—H4*A*···N3^iii^	0.96	2.63	3.436 (3)	142
C5—H5*A*···N3^iv^	0.96	2.64	3.586 (3)	169

Symmetry codes: (i) −*x*+1, −*y*+1, −*z*+1; (ii) −*x*+1, *y*, −*z*+3/2; (iii) *x*−1/2, *y*+3/2, *z*; (iv) *x*−1/2, *y*+1/2, *z*.
